# RgIA4 Prevention of Acute Oxaliplatin-Induced Cold Allodynia Requires α9-Containing Nicotinic Acetylcholine Receptors and CD3^+^ T-Cells

**DOI:** 10.3390/cells11223561

**Published:** 2022-11-11

**Authors:** Peter N. Huynh, Sean B. Christensen, J. Michael McIntosh

**Affiliations:** 1 School of Biological Sciences, University of Utah, Salt Lake City, UT 84112, USA; 2 George E. Wahlen Veterans Affairs Medical Center, Salt Lake City, UT 84112, USA; 3 Department of Psychiatry, University of Utah, Salt Lake City, UT 84112, USA

**Keywords:** α9-containing, nAChR, cold allodynia, T-cells, analgesia, non-opioid

## Abstract

Chemotherapy-induced neuropathic pain is a debilitating and dose-limiting side effect. Oxaliplatin is a third-generation platinum and antineoplastic compound that is commonly used to treat colorectal cancer and commonly yields neuropathic side effects. Available drugs such as duloxetine provide only modest benefits against oxaliplatin-induced neuropathy. A particularly disruptive symptom of oxaliplatin is painful cold sensitivity, known as cold allodynia. Previous studies of the *Conus regius* peptide, RgIA, and its analogs have demonstrated relief from oxaliplatin-induced cold allodynia, yielding improvement that persists even after treatment cessation. Moreover, underlying inflammatory and neuronal protection were shown at the cellular level in chronic constriction nerve injury models, consistent with disease-modifying effects. Despite these promising preclinical outcomes, the underlying molecular mechanism of action of RgIA4 remains an area of active investigation. This study aimed to determine the necessity of the α9 nAChR subunit and potential T-cell mechanisms in RgIA4 efficacy against acute oxaliplatin-induced cold allodynia. A single dose of oxaliplatin (10 mg/kg) was utilized followed by four daily doses of RgIA4. Subcutaneous administration of RgIA4 (40 µg/kg) prevented cold allodynia in wildtype mice but not in mice lacking the α9 nAChR-encoding gene, *chrna9*. RgIA4 also failed to reverse allodynia in mice depleted of CD3^+^ T-cells. In wildtype mice treated with oxaliplatin, quantitated circulating T-cells remained unaffected by RgIA4. Together, these results show that RgIA4 requires both *chrna9* and CD3^+^ T-cells to exert its protective effects against acute cold-allodynia produced by oxaliplatin.

## 1. Introduction

Chemotherapy-induced peripheral neuropathy (CIPN) is a common side effect of several first-line anti-cancer drugs that leads to long-term pathologies in 20–30% of patients. Currently, there are no FDA-approved therapeutics for the effective treatment or prevention of CIPN [1,2]. These neuropathic side effects can be dose-limiting and may generate a long-lasting need for pain management that can profoundly affect a patient’s quality of life [3]. Therefore, there is an urgent need for adjuvant therapies to alleviate CIPN, focused on improving patient outcomes and their subsequent quality of life.

Among chemotherapeutics, the prevalence of oxaliplatin-induced peripheral neuropathy (OIPN) has resulted in its frequent use to study neuropathic pain in rodents. The platinum-containing anti-cancer drug is widely used to treat colorectal cancer yet produces both acute and chronic neuropathic pain in both human patients and rodent models [4]. Thus, there has been a substantial effort toward understanding the neurological and inflammatory changes involved in its complex suite of symptoms. However, a comprehensive mechanism has not yet been elucidated.

One of the most common symptoms of OIPN is painful cold sensitivity, termed cold allodynia. Cold allodynia manifests in both acute and chronic dosing regimens of oxaliplatin and can either resolve quickly after treatment cessation or persist long after [5]. Due to the sensory pathologies from oxaliplatin, many neuronal effects have been studied. Surface expression and excitability changes in cold-sensing and pain-sensing neurons have been observed [6,7]. In parallel, immunomodulatory effects of oxaliplatin were investigated, showing distinct shifts in T- and B-cell populations after dosing [8]. Notably, the infiltration and accumulation of peripheral immune cells into perineural spaces of injured neurons have been posited as associated steps in developing and maintaining chronic neuropathic pain [9]. While cold allodynia may manifest similarly in both acute and chronic models, important cellular and molecular changes differ and should be considered when designing a targeted therapeutic adjuvant. To focus on the role of T-cells in the initiation of cold allodynia, an acute oxaliplatin model was employed.

OIPN can be reliably induced in many strains of laboratory rodents, yielding a diversity of symptoms based on the dosing regimens and strains tested [10]. Using some of these models, we have previously shown the efficacy of α9α10 nicotinic acetylcholine receptor (nAChR) antagonists in the prevention and resolution of cold allodynia [11,12]. These studies accompany a larger body of work that has established α9α10 nAChRs as a target for treating chronic pain across several distinct models of neuropathic pain [13,14].

The characterization of α9-containing nAChR subtypes was accelerated by the refinement of naturally selective peptides isolated from the venoms of carnivorous cone snails. Namely, the short peptide RgIA isolated from the worm-hunting *Conus regius* was identified as a naturally selective antagonist of ionic currents in rodent α9-containing nAChRs. This peptide was amenable to amino acid substitutions to tune its potency and selectivity for human receptors [11,15,16,17,18,19,20]. These refinements produced the second-generation analog, RgIA4, which was optimized for potency and selectivity for α9α10 nAChRs [11]. RgIA and RgIA4, alleviated pain in several models of neuropathic pain. Moreover, treatment with RgIA4 in a 3-week chronic oxaliplatin regimen yielded pain relief that persisted several weeks beyond their dosing regimen [11,21]. At the cellular level, RgIA yielded protective effects on neurons and glia in rats, preventing reduction in nerve fibers, myelin thickness, and axon diameter, correlated with improved behavioral scores in mechanical allodynia assays [22]. Using constitutive knockouts confirmed that the α9-encoding gene, *chrna9*, is required for RgIA4 to confer its therapeutic efficacy [11]. α9 subunits generally combine with α10 subunits and may also combine with α7 subunits [23,24]. As the combinatorial assembly of nAChRs are complex and various stoichiometries may continue to be discovered, we refer to the potential target of RgIA and its analogs as α9-containing nAChRs. These underlying physiological changes and long-term relief from chronic neuropathic pain suggest a fundamental disease-modifying effect from α9-containing nAChR modulation by RgIA and its analogs.

Mechanistically, the exact cellular and physiological targets of RgIA and its analogs have not yet been elucidated. Due to the efficacy of RgIA4 in both chemotherapy and physical trauma models of neuropathic pain, the mechanism of action appears to occur at a convergent step of chronic pain development. However, α9 and α10 nAChR subunits are notably absent in the brain, only expressed in a small subset of human nociceptors in the dorsal root ganglia (DRG), and also were not detected in mouse DRG nociceptors, suggesting that the activity at these receptors does not directly blunt the sensation of pain [25,26,27].

Transcripts for α9 and α10 subunits have been found in purified T-cells, B-cells, and immortalized T-cell lines (CEM, Jurkat, and MT2) [28]. The α9-encoding gene, *chrna9*, is upregulated in CD4^+^CD8^+^ double-positive (DP) thymocytes, as well as late-stage induced regulatory T-cells (iT_regs_) [29,30]. However, acetylcholine-elicited ionic responses have not been observed from α9-containing nAChRs in T-cells, suggesting a possible non-canonical role of these receptors in modulating immune responses. α-Conotoxin binding to α9-containing nAChRs has also been shown to affect the release of pro-inflammatory cytokines, and infiltration of immune cells around injured nerves [22,31,32]. These data suggest a diversity of immune effects mediated by α9-containing nAChRs, potentially contributing to a balancing feedback role in maintaining inflammatory homeostasis.

In the context of peripheral nerve injury, neuroinflammation is considered an important associated step in developing neuropathic pain [33]. T-cells are varied in function, playing both protective and exacerbating roles in neuroinflammation and chronic pain [9,33,34,35]. While α9-containing nAChRs may have differing roles across several distinct immune cell types, due to the restricted expression profile and systemic roles of T-cells in neuroinflammatory pain, the present study focused on peripheral T-cells.

## 2. Materials and Methods

### 2.1. Acute Oxaliplatin-Induced Cold Allodynia

Oxaliplatin (Cat. HY-17371; MedChem Express, Monmouth Junction, NJ, USA) was dissolved at 2.5 mg/mL in 0.22 µm-filtered 0.9% sodium chloride (NaCl) saline solution. The test compound, RgIA4, was dissolved at 10 µg/mL in filtered saline. Mice were injected with either saline (intraperitoneally; i.p.) or oxaliplatin (10 mg/kg, i.p.) in a single administration, followed by daily injections of either saline (subcutaneously; s.c.) or RgIA4 (40 µg/kg; s.c.). Treatment regimens were started on Mondays (day 1), allowing for consecutive injections for 4 days preceding behavioral and hematological analysis.

### 2.2. CD3^+^ Cell Depletion

The depletion of CD3^+^ T-cells was implemented by intraperitoneally pre-treating mice with a low-endotoxin anti-mouse CD3ε polyclonal IgG (2.0 mg/kg; Cat. BE0001-1, Bio X Cell, Lebanon, NH, USA) on days −4 and −2. Maintenance of CD3^+^ cell depletion was measured via a terminal blood draw via cardiac puncture followed by flow cytometry analysis. Detailed depletion antibody product details are shown in Table S1.

### 2.3. Scoring of Cold Allodynia in Mice

Mice were assessed for cold allodynia via a cold plate assay. Baseline readings were established before induction of cold allodynia on day 1, and on day 5, approximately 24 h after the final drug treatment. Briefly, mice were placed in an incremental hot/cold plate test chamber (IITC, Inc Life Science, Woodland, CA, USA) and allowed to acclimate at room temperature (23 °C) for 5 min. The temperature-regulated chamber floor was then cooled at a rate of 10 °C per min. The testing was stopped when the animal lifted both forepaws and shaking or licking occurred as a sign of nocifensive behavior. Alternating lifting of forepaws was not scored. Throughout the study period, experimenters were blinded to the identity of the injected compounds. Mice were preconditioned at least once prior to acquiring baseline readings.

### 2.4. Flow Cytometry of Peripheral White Blood Cells (PWBCs)

All labeled antibodies used in this study and their immunostaining concentrations are shown in Table S1. Mice were anesthetized using CO_2_ and further euthanized via cervical dislocation immediately preceding blood collection. Whole blood was collected using a terminal cardiac puncture through a “21 G × 1” needle into an EDTA (0.5 M) treated 3cc syringe. Approximately 0.5–1.0 mL of whole blood was withdrawn per mouse.

Working aliquots of 100 µL of whole blood were lysed in 15× volume of ammonium-chloride-potassium (ACK) red blood cell (RBC) lysis buffer (ammonium chloride [155 mM], sodium bicarbonate [10 mM], ethylenediaminetetraacetic acid (EDTA) [125 µM], pH 7.4) for 10 min at room temperature and pelleted at 500× *g* for 10 min for two total rounds of lysis. The RBC-cleaned PWBCs were washed in 1.5 mL of Dulbecco’s calcium- and magnesium-free phosphate-buffered saline (DPBS) and pelleted at 500× *g* for 10 min at room temperature.

The washed PWBCs were resuspended into 100 µL of Fixable Viability Stain 575 (BD Horizon) at 0.59 µg/test; the working solution was made in FBS-free DMEM media (Penicillin (100 IU), Streptomycin (100 μg/mL)). Singly labeled PWBCs were washed and pelleted at 500× *g* for 10 min at room temperature. Pellets were resuspended in 100 µL of antibody cocktail diluted in fully supplemented DMEM media [Penicillin (100 IU), Streptomycin (100 µg/mL), fetal bovine serum (FBS; 10% *v*/*v*)], followed by incubation at room temperature in the dark for 1 h. Cells were washed twice in DPBS and stored in a dark container for transfer to the flow cytometry instrument. 50,000 events were collected using a 4-laser, 9-color CytoFLEX Research Flow Cytometer (Beckman Coulter Life Sciences, Indianapolis, IN, USA) and analyzed using FlowJo V.10.8.0 software (FlowJo, Ashland, OR, USA).

### 2.5. Animals

Wildtype CBA/CaJ mice were obtained from Jackson Labs (Bar Harbor, ME, USA). Germline *chrna9* knockout (KO) mice originally on a 129Sv/Ev and CBA/CaJ background were crossed using an accelerated backcrossing program (Jackson Labs) until 99.5% identity with wildtype CBA/CaJ mice was achieved [36]. Mice were then further backcrossed with wildtype CBA/CaJ mice for an additional three generations. Adult mice for the current study were obtained from actively maintained in-house colonies.

## 3. Results

### 3.1. Induction of Acute Oxaliplatin Cold Allodynia in CBA/CaJ Mice

**Figure 1:**
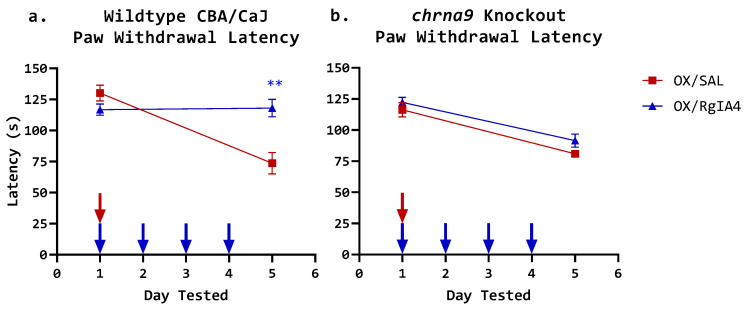
Acute oxaliplatin-induced cold allodynia is prevented by RgIA4 (s.c., 40 µg/kg) in wildtype mice but not in *chrna9* knockout mice. A single dose of oxaliplatin (OX, i.p., 10 mg/kg) on day 1 was used to induce cold allodynia as described in Materials and Methods. Paw withdrawal latencies were obtained prior to oxaliplatin treatment on day 1, and approximately 24 h after the final drug administration on day 5. (**a**) Oxaliplatin-induced cold allodynia is manifested as a decreased paw withdrawal latency in wildtype CBA/CaJ mice. Cold allodynia is prevented by treatment with RgIA4, but not in mice that received subcutaneous saline (SAL) as a control. (**b**) RgIA4 does not prevent cold allodynia in *chrna9* knockout mice, indicating the necessity of α9-containing nAChRs for therapeutic effect. Arrows indicate administration of oxaliplatin (red, top) or RgIA4 (blue, bottom). Paw withdrawal latencies are depicted as the mean time in seconds ± SEM (*n* = 8). Asterisks (*) denote significance compared to the OX/SAL control. The level of significance is indicated as *p* ≤ 0.01, **, determined using an unpaired two-tailed *t*-test.

To model oxaliplatin-induced cold allodynia, the platinum-containing chemotherapeutic drug oxaliplatin was utilized. Adult CBA/CaJ mice were administered a single intraperitoneal (i.p.) injection of saline (SAL) or oxaliplatin (OX; 10 mg/kg) on day 1 and a subcutaneous (s.c.) injection of either saline or RgIA4 (40 µg/kg). Subcutaneous treatments with RgIA4 or saline were continued once daily for the duration of the treatment protocol (days 1–4). Oxaliplatin treatment in the absence of RgIA4 reliably induced cold allodynia, a characteristic feature of oxaliplatin-induced neuropathic pain. The induction of cold allodynia was measured by a paw withdrawal assay, utilizing a temperature-regulated cold plate chamber in which the floor was cooled at 10 °C/min. Paw withdrawal latencies were obtained prior to induction of cold allodynia on day 1 and on day 5 approximately 24 h after the final drug administration. Testing on day 5 (four days after oxaliplatin dosage) showed cold nocifensive behavior was elicited in the OX/SAL treatment group compared to their pre-treatment behavior. The mean paw withdrawal latency decreased from 130.2 s (SEM = 6.4 s) before treatment to 73.7 s (SEM = 8.7 s) on day 5 (*p* = 0.0001, ***, unpaired two-tailed *t*-test day 1 vs. day 5). This cold allodynia was effectively prevented by daily dosages of RgIA4 (40 µg/kg, s.c.), maintaining the paw withdrawal latency at 118.1 s (SEM = 7.0 s) on day 5 (Figure 1a; *p* = 0.0014, **; unpaired two-tailed *t*-test OX/SAL vs. OX/RgIA4). Therefore, acute cold allodynia from oxaliplatin was induced in wildtype CBA/CaJ mice and successfully prevented by administration of RgIA4.

To investigate the necessity of α9-containing nAChRs in RgIA4 efficacy against acute cold allodynia, the same regimen was tested using *chrna9* knockout mice [22]. On day 5, four days after the administration of oxaliplatin (10 mg/kg; IP), the development of cold allodynia was successfully induced in α9 subunit KO mice. The mean paw withdrawal latency decreased from 116.4 s (SEM = 6.4 s) before treatment to 80.9 s (SEM = 2.9 s) on day 5 (Figure 1b; *p* < 0.001, ***; unpaired two-tailed *t*-test day 1 vs. day 5). However, the behavioral rescue by RgIA4 was not observed in *chrna9* knockout mice, in which the mean paw withdrawal latency decreased from 122.4 s (SEM = 4.0 s) to 91.6 s (SEM = 5.3 s) after oxaliplatin treatment (Figure 1b; *p* = 0.0004, ***; unpaired two-tailed *t*-test day 1 vs. day 5). The behavioral output did not significantly differ between OX/SAL and OX/RgIA4 mice on day 5 (*p* = 0.096, ns; unpaired two-tailed *t*-test). The reduced paw withdrawal latencies between days 1 and 5 in *chrna9* knockout mice show that germine deletion of α9-containing nAChRs does not prevent the onset of oxaliplatin-mediated cold allodynia. However, the loss of RgIA4-mediated pain relief in *chrna9* knockout mice indicates that α9-containing nAChRs are a necessary target for RgIA4 efficacy in alleviating oxaliplatin-induced cold allodynia in this acute model.

### 3.2. CD3^+^ T-Cell Depletion Prevents RgIA4 Rescue in Acute Cold Allodynia

**Figure 2:**
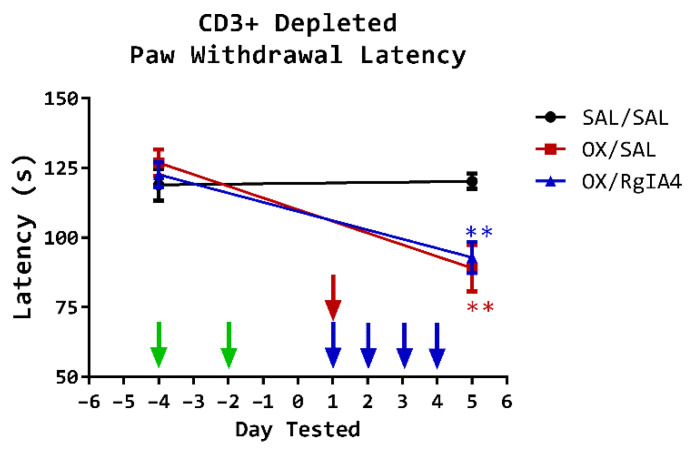
CD3^+^ cell depletion abolishes RgIA4-mediated analgesia. All mice were depleted of CD3+ T-cells using a low endotoxin anti-mouse CD3ε IgG1 antibody as described in Materials and Methods. Subsequently, mice were injected i.p. with either saline (SAL) as control or oxaliplatin (OX, 10 mg/kg). Oxaliplatin-induced cold allodynia is indicated by a decrease in paw withdrawal latency. In the absence of CD3+ cells, RgIA4 (40 µg/kg s.c.) failed to prevent oxaliplatin-induced cold allodynia. Treatment with anti-mouse CD3ε antibody is shown on days −4 and −2 (green arrows), oxaliplatin treatment is indicated on day 1 (red arrow, top), and RgIA4 treatment is shown on days 1–4 (blue arrows, bottom). Paw withdrawal latencies are depicted as the mean time in seconds ± SEM (SAL/SAL *n* = 9, OX/SAL *n* = 9, and OX/RgIA4 = 10). Asterisks (*) denote significance compared to SAL/SAL. The level of significance is indicated as *p* ≤ 0.01, ** Significance was determined using an ordinary one-way ANOVA followed by Tukey’s multiple comparisons test.

To investigate the role of T-cell-expressed α9-containing nAChRs in the prevention and resolution of oxaliplatin-induced cold allodynia, CD3^+^ T-cells were depleted using an anti-mouse CD3 monoclonal antibody [37]. Prior to cold allodynia induction, mice were intraperitoneally administered In Vivo MAb Armenian hamster anti-mouse CD3ε IgG1 monoclonal antibody (2.0 mg/kg, i.p.; BioXCell cat. BE0001-1; clone 145-2C11) on days −4 and −2. No adverse effects were noted. Paw withdrawal latencies decreased from 126.9 s (SEM = 4.8 s) pretreatment to 89.0 s (SEM = 8.4 s) after oxaliplatin treatment in OX/SAL treated mice (*p* = 0.0012, **, unpaired two-tailed *t*-test). This cold allodynia was not prevented by the coadministration of RgIA4, with latencies dropping from 122.6 s (SEM = 4.4 s) pretreatment to 92.9 s (SEM = 5.6 s) after oxaliplatin treatment. The depletion of CD3^+^ cells prior to oxaliplatin treatment eliminated the protective effects of RgIA4 as measured on day 5 of the testing regimen (Figure 2; *p* = 0.89, ns; one-way ANOVA). Thus, CD3^+^ cells are necessary for RgIA4-mediated analgesia.

### 3.3. Peripheral White Blood Cell (PWBC) Changes Due to Oxaliplatin and RgIA4

**Figure 3:**
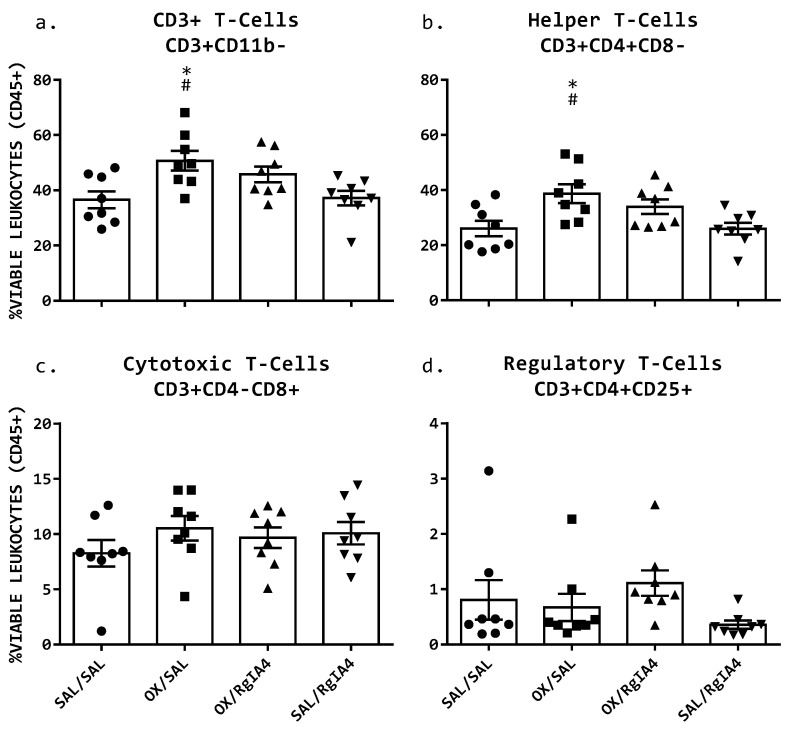
Peripheral white blood cell (PWBC) analysis by flow cytometry. Peripheral whole blood was collected via terminal cardiac puncture on day 5. Mice received an intraperitoneal dosage of either saline (SAL) or oxaliplatin (OX, 10 mg/kg) on day 1, followed by four daily doses of either saline or RgIA4 (40 μg/kg). (**a**) CD3^+^ T-cells and (**b**) CD3^+^CD4^+^ helper T-cells show an increased circulating population in oxaliplatin-treated OX/SAL mice. This elevation is not prevented by coadministration with RgIA4 in OX/RgIA4 mice. No major population shifts were observed in (**c**) cytotoxic T-cells (CD3^+^CD8^+^) or (**d**) regulatory T-cells (CD3^+^CD4^+^CD25^+^). Populations are reported as mean %viable leukocytes (CD45^+^) ± SEM (*n* = 8). Asterisks (*) denote significance compared to SAL/SAL. Hashes (#) denote significance compared to SAL/RgIA4. The level of significance is indicated as *p* ≤ 0.05, * Significance was determined using an ordinary one-way ANOVA followed by Tukey’s multiple comparisons test.

**Figure 4:**
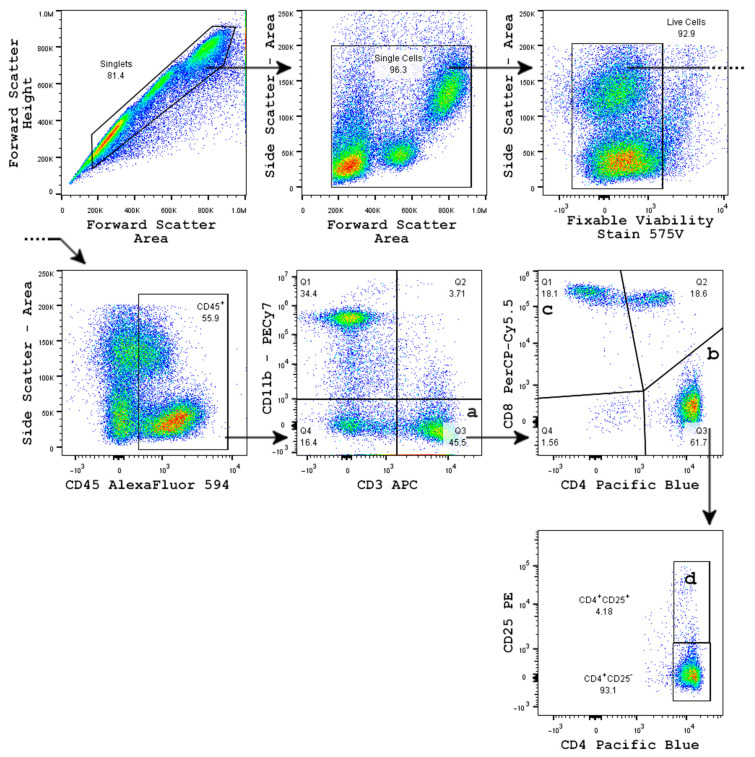
Flow cytometry gating scheme to identify subpopulations of peripheral white blood cells (PWBCs). Populations quantitated are circulating (a) collective T-cells (CD3^+^), (b) helper T-cells (CD3^+^CD4^+^), (c) cytotoxic T-cells (CD3^+^CD8^+^), and (d) regulatory T-cells (CD3^+^CD4^+^CD25^+^).

Circulating peripheral white blood cells were cleaned of red blood cells and quantitated as a percentage of viable, circulating CD45^+^ leukocytes. An increase in CD3^+^ T-cells was observed in response to oxaliplatin treatment (Figure 3a), rising from 36.6% (SEM = 3.08) in SAL/SAL treated mice to 50.7% (SEM = 3.53) in OX/SAL-treated mice (* *p* = 0.014 one-way ANOVA). This oxaliplatin-induced T-cell elevation was not prevented by coadministration of RgIA4 in the OX/RgIA4 treatment group (45.8%, SEM = 2.89; ns, *p* = 0.17 one-way ANOVA vs. SAL/SAL mice). The circulating T-cell proportion was not affected by RgIA4 treatment alone, with SAL/RgIA4-treated mice exhibiting a CD3^+^ cell proportion of 37.2% (SEM = 2.62; ns, *p* = 0.999, one-way ANOVA vs. SAL/SAL mice), Figure 3. Moreover, the elevation of T-cells was specifically reflected in helper T-cells (CD3^+^CD4^+^), shown in Figure 3b. Helper T-cells increased from 26.0% (SEM = 2.82) in SAL/SAL mice to 38.6% (SEM = 3.43) in OX/SAL mice (*, *p* = 0.0179 one-way ANOVA vs. SAL/SAL mice). Overall CD3^+^ T-cell proportions and CD3^+^CD4^+^ helper T-cells elevations were not altered by OX/RgIA4 treatment (34.0%, SEM = 2.67; ns, *p* = 0.214 one-way ANOVA vs. SAL/SAL mice). No significant elevation or depletion was observed in cytotoxic T-cells (CD3^+^CD8^+^), regulatory T-cells (CD3^+^CD4^+^CD25^+^), as shown in Figure 3c,d. Gating and determination of populations are shown in Figure 4.

## 4. Discussion

α-Conotoxins RgIA and Vc1.1 (specific α9-containing nAChR ligands) have demonstrated analgesic activity in several animal models of neuropathic pain including chronic oxaliplatin-induced neuropathic pain [11,32,38,39,40]. The side effects of oxaliplatin can manifest as acute and/or chronic symptoms. RgIA4 is an analog of the *Conus regius* RgIA peptide, which was optimized for potency against the human α9α10 nAChR and has little or no activity at other pain-relevant targets [11]. Previous work demonstrated the efficacy of RgIA4 in preventing neuropathic pain resulting from chronic administration of oxaliplatin [11,21,41]. The present study adds to this body of knowledge by demonstrating the efficacy of RgIA4 in a 4-day acute oxaliplatin dosing regimen.

Several studies have indicated that certain α-conopeptides act as agonists of the GABA_B_ receptor resulting in analgesic effects [42,43,44,45]. This has led to questions regarding the role and necessity of α9-containing nAChRs in mediating α-conopeptide analgesia. By employing α9 nAChR subunit (*chrna9*) knockout mice, we determined that acute therapeutic effects of RgIA4 in oxaliplatin-induced cold-allodynia require α9-containing nAChRs. These findings are consistent with those reported for RgIA-5524, a methylene thioacetal analog of RgIA; as in the present study, subunit deletion of the α9 nAChR subunit abolished analgesic activity [20]. Thus, while GABA_B_ agonist effects contribute to the analgesic activity of some α-conopeptides, the α9 nAChR subunit is necessary for the activity of at least two of the RgIA analogs that have been optimized for potency at the human α9α10 nAChR. It is of interest that cold allodynia is reliably induced with a single dose of oxaliplatin treatment in both wildtype and *chrna9* knockout animals. Thus, mice with germline deletion of the α9 subunit differ in the development of allodynia compared to mice where the function of the α9-containing receptor is transiently blocked. Alternatively, the binding of RgIA4 to α9-containing nAChRs may itself induce conformational changes in the receptor that, in a metabotropic-like function, influence the functioning of neighboring signaling molecules.

Although the necessity of α9-containing nAChRs in RgIA4 analgesia is evident, the physiological mechanism of action remains unknown. α9-containing nAChRs have a particularly restricted expression profile, notably being absent in the brain [25,26]. The highly charged nature of RgIA and RgIA4 likely also prevents penetration of the blood–brain barrier. Moreover, RgIA4 produced long-lasting pain relief that persisted at least three weeks beyond treatment cessation in a chronic oxaliplatin model, reflecting an underlying modification of the disease state [21]. The native peptide, RgIA preserved nerve fiber counts and myelin sheath thickness in a physical chronic constriction injury model [22]. The long-term pain resolution by RgIA and RgIA4, along with the absence of α9-containing nAChRs in the central nervous system indicate that its primary effects do not lie in blunting central sensations of pain. Together, these findings indicate that RgIA4 acts peripherally to produce its long-term chronic pain relief via disease-modifying effects.

The restricted expression profile of α9-containing nAChRs and several previous findings with RgIA analogs implicate immune cells, namely lymphocytes, as one possible site of action for RgIA4. α9 and α10 nAChR transcripts have been measured in T- and B- lymphocytes; however, their function in these cells has not been fully characterized [28]. Non-canonical signaling is implicated, in part, due to the lack of measurable nAChR-mediated ionic currents in these cells. [28] Although the signaling of α9-containing nAChRs in lymphocytes remains under investigation, the expression of *chrna9* has been shown to be downregulated upon transition of naïve T-cells to T_h_1 and T_h_2 helper T-cells and upregulated after transition to T_h_17 and regulatory T-cells [30]. In monocytic cell lines, RgIA4 was posited to affect the release of cytokines via modulation of P2 × 7 purinergic receptors and inflammasome assembly. [46] In lymphocytes, a detailed signaling mechanism of α9-containing nAChRs has yet to be elucidated. However, α9-containing nAChRs may play a role in T- and B-cell activation [47].

T-cells, in particular, have been implicated in both the exacerbation and resolution of neuropathic pain [9,35,48,49]. While the primary effect of α9-containing nAChRs in T-cells has not been comprehensively studied, the parent peptide, RgIA, was shown to reduce the infiltration of lymphocytes and macrophages to sites of nerve injury in vivo in a chronic constriction nerve injury model [32]. The necessity of T-lymphocytes in RgIA4-mediated pain resolution was demonstrated in the current study, by the loss of RgIA4 efficacy when CD3^+^ cells were depleted.

While T-cells are prominent effector cells, they also play a central role in cellular and cytokine signaling. Helper T-cells have been shown to modulate macrophage polarization dynamics between M1 (primarily pro-inflammatory) and M2 (primarily anti-inflammatory) states by altering cytokine microenvironments [50]. Endogenous opioid release from T-cells has also been shown to directly blunt the sensation of pain [51]. However, the long-lasting relief caused by RgIA4 treatment suggests this is not the primary pathway of interaction. α9-containing nAChRs have been shown to play direct roles in modulating cytokine release. RgIA4 directly and positively modulated the release of the pro-inflammatory cytokine, IL-1β, in a monocytic cell line [31]. In contrast, RgIA also increased cellular release of the potent anti-inflammatory cytokine, IL-10, from isolated granulocytes, increased their cell adhesiveness, and reduced reactive oxygen species in these cells [52]. Despite opposing effects in different cell types, the net in vivo effects of RgIA and RgIA4 are anti-inflammatory and analgesic, providing long-lasting relief and neuronal protection. Together, these cellular effects of α9-containing nAChR interactions suggest RgIA4 likely acts on more than one immune cell type. The diversity of immune effects from RgIA4 also suggests a balancing feedback role of α9-containing nAChRs, yielding both pro- and anti-inflammatory effects, perhaps depending on the local cellular environment.

The unchanged circulating counts of CD3^+^ and CD3^+^CD4^+^ cells observed in the current study suggest that the primary driver of RgIA4-mediated relief is not simply an altered production or mobilization of T-cells within these mice. Thus, the anti-inflammatory effects of RgIA4 may lie within more nuanced cellular processes such as modulating cytokine signaling. Identification of CD3^+^ T-cells as a necessary population for RgIA4 efficacy adds further insight into the role of α9-containing nAChR mechanisms in providing pain relief and neuronal protection. The *chrna9* gene is upregulated within several distinct developmental stages of T-cells, including early double-positive (CD4^+^CD8^+^) thymocytes in the thymus and late-induced regulatory T-cells [29,30]. The presence of both *chrna9* and *chrna10* transcripts was also observed in mature T- and B-cells [28]. These transcriptome data, in combination with the necessity of CD3^+^ T-cells, warrant further research into the specific roles and effects of α9-containing nAChR signaling in these immune cells. Future studies to determine the specific involvement of the various T-cell subpopulations, including subtypes of helper T-cells, and B-cells may refine our understanding of CD3+ cell-dependent allodynia. Characterizing changes in cellular signaling, activation, anergy, and secreted inflammatory cytokines by α9-containing nAChRs in these cells may additionally aid understanding the role of CD3+ T-cells in this non-opioid based pain relief.

## 5. Conclusions

In this study, we show that α9-containing nAChRs and CD3^+^ T-cells are necessary for RgIA4 protection against acute oxaliplatin neuropathic pain via a non-opioid mechanism. The unchanged circulating T-cell count combined with the necessity of CD3^+^ T-cells indicates that RgIA4 outcomes depend on T-cell signaling mechanisms, rather than increased production or circulation of T-cells. The effects of modulating α9-containing nAChRs within specific T-cell subsets may produce different mechanistic outcomes within cellular microenvironments and warrant further study to fully characterize these dynamics within tissue-specific niches.

## Data Availability

Not applicable.

## Supplementary Materials

The following supporting information can be downloaded at: https://www.mdpi.com/article/10.3390/cells11223561/s1, Table S1: Commercial antibodies used in flow cytometry analysis and CD3^+^ cell depletion accompanied with test concentrations.
