# Supplementary material for: RgIA4 Prevention of Acute Oxaliplatin-Induced Cold Allodynia Requires α9-Containing Nicotinic Acetylcholine Receptors and CD3+ T-Cells
Source: Cells. 2022 Nov 11;11(22):3561. doi: 10.3390/cells11223561 (PMC9688540; doi:10.3390/cells11223561)
Supplement: Supplementary file 1 [file cells-11-03561-s001.zip › cells-1960635-supplementary.pdf]

cells

MDPI

Article

RgIA4 Prevention of Acute Oxaliplatin-Induced Cold Allodynia Requires α9-Containing Nicotinic Acetylcholine Receptors and CD3+ T-Cells

Peter N. Huynh [1,*], Sean B. Christensen [1] and J. Michael McIntosh [1,2,3]

[1] School of Biological Sciences, University of Utah, Salt Lake City, UT 84112, USA
[2] George E. Wahlen Veterans Affairs Medical Center, Salt Lake City, UT 84112, USA
[3] Department of Psychiatry, University of Utah, Salt Lake City, UT 84112, USA
* Correspondence: peter.huynh@utah.edu

Supplemental Information

Table S1: Commercial antibodies used in flow cytometry analysis and CD3+ cell depletion accompanied with test concentrations.

**Flow Cytometry Antibodies**								
**Target**	**Fluorophore**	**Clone**	**µg/test**	**Host**	**Reactivity**	**Isotype**	**Manufacturer**	**Catalog No.**
Viability	FVS575V	N/A	0.59	N/A	N/A	N/A	BD Horizon	565694
CD45	AlexaFluor 594	30-F11	0.25	Rat	Mouse	IgG2c	Bio-Rad	128011
CD11b	PE/Cyanine7	M1/70	0.625	Rat	Mouse	IgG2b	Invitrogen	25-01122-81
CD3	APC	KT3	10	Rat	Mouse	IgG2a	Bio-Rad	MCA500APC
CD4	Pacific Blue	RM4-5	10	Rat	Mouse	IgG2a	Bio-Rad	MCA2691PB
CD8	PE-Cy5.5	53.6.7	0.625	Rat	Mouse	IgG2a κ	ThermoFisher	35-0081-80
CD25	PE	PC61.5.3	5	Rat	Mouse	IgG1	ThermoFisher	MA5-17817
***In vivo* depletion antibodies**								
**Target**	**Fluorophore**	**Clone**	**mg/kg**	**Host**	**Reactivity**	**Isotype**	**Manufacturer**	**Catalog No.**
CD3ε	-	145-2C11	1000	Armenian Hamster	Mouse	IgG1	BioXCell	BE0001-1
